# Combination Therapy for Overcoming Multidrug Resistance in Breast Cancer Through Hedgehog Signaling Pathway Regulation

**DOI:** 10.3390/pharmaceutics17050572

**Published:** 2025-04-26

**Authors:** Yujie Liu, Yiliang Yang, Xianrong Qi

**Affiliations:** 1 Department of Pharmaceutics, School of Pharmaceutical Sciences, Peking University, Beijing 100191, China; liuyujiemao@126.com (Y.L.); yiliangy@buu.edu.cn (Y.Y.); 2 College of Biochemical Engineering, Beijing Union University, Beijing 100023, China

**Keywords:** sonic hedgehog pathway, multidrug resistance, cyclopamine, nano-drug delivery system, combination therapy

## Abstract

**Background/Objectives:** The ineffective delivery of drugs into tumors and the existence of multidrug resistance (MDR) are the primary causes of chemotherapy failure. Downregulation of the Sonic Hedgehog (Shh) pathway has been shown to reduce P-glycoprotein (P-gp) expression on cell membranes and to resist MDR. **Methods:** In this study, we combine cyclopamine (CYP, a potent Shh antagonist) with paclitaxel (PTX, an antitumor drug that can produce MDR) in a nano-drug delivery system (CYP NP and PTX NP) for the treatment of drug-resistant breast cancer. Nanoparticles were characterized for size, zeta potential, and encapsulation efficiency. P-gp expression, nanoparticle accumulation, cytotoxicity, and apoptosis were evaluated in MCF-7 and MCF-7/Adr cells. Penetration ability was assessed using 3D multicellular tumor spheroids. Antitumor efficacy and nanoparticle biodistribution were validated in MCF-7/Adr-bearing nude mice models. **Results:** Our engineered CYP nanoparticles (~200 nm) demonstrated prolonged intratumoral retention, enabling sustained Shh pathway inhibition and P-gp functional suppression. This size-optimized formulation created a favorable tumor microenvironment for the smaller PTX nanoparticles (~30 nm), facilitating deeper tumor penetration and enhanced cellular uptake. Meanwhile, by down-regulating P-gp expression, CYP NPs could convert drug-resistant cells to PTX-sensitive cells in both cytotoxicity and apoptosis induction through the Shh pathway. The combination of CYP NP and PTX NP augmented the antitumor effects in MCF-7/Adr-bearing nude mice models. **Conclusions:** The CYP NP and PTX NP combination offers a new therapeutic strategy in cancer treatment.

## 1. Introduction

According to the latest cancer data released by the World Health Organization, the number of new cases of breast cancer (2.26 million) has surpassed lung cancer (2.2 million) to become the most common cancer in the world [1]. Chemotherapeutic agents are extensively utilized in the therapeutic treatment of malignant tumors. However, multidrug resistance (MDR) has become a significant impediment to its efficacy. The problem is particularly acute in the area of breast cancer. MDR is usually induced by long-term chemotherapy, and its molecular mechanism is complex, involving multiple factors such as enhanced function of drug efflux pumps (such as the ABC transporter family), altered drug metabolism pathway, and evasion of apoptosis [2,3]. Among them, drug efflux caused by the overexpression of ABC transporters (such as P-gp) is recognized as one of the major mechanisms of MDR [4,5]. Studies have shown that the expression of ABC transporters is regulated by multiple signaling pathways [6]. Therefore, targeting these pathways may provide novel strategies for reversing MDR.

The Sonic Hedgehog (Shh) signaling pathway is a highly conserved molecular cascade essential for embryonic development, tissue regeneration, and stem cell regulation [7,8]. In recent years, its association with MDR has been gradually revealed and has emerged as a promising target for cancer therapy [9]. Consequently, research focus has been directed to develop Shh pathway-specific therapeutics for application in cancer treatment and research [10]. Representative of such breakthroughs is the development of the FDA-approved Shh inhibitors vismodegib and erismodegib for Smoothened (Smo) [11,12]. Studies have also shown that the Shh pathway is not only a vital pathway regulating cell proliferation and differentiation but also promotes MDR through direct activation of ABC transporter transcription [13]. Structurally, the pathway is initiated by the Shh ligand, which binds to its transmembrane receptor Patched 1 (PTCH1), relieving PTCH1-mediated suppression of the G protein-coupled receptor Smoothened (SMO). Activated SMO triggers a downstream signaling cascade that culminates in the nuclear translocation of Gli transcription factors (Gli1, Gli2, Gli3), which modulate the expression of target genes such as ABC transporters [14,15]. It is worth noting that a number of studies have confirmed that the Shh-Gli axis can directly bind the promoter region of ABC transporters (such as ABCB1/P-gp, ABCC1/MRP1) and enhance their transcriptional activity [16,17]. Furthermore, Shh overexpression is associated with the generation and sustainment of cancers, including glioblastoma, breast cancer, and chronic myeloid leukemia (CML) [7]. Therefore, targeting the Shh pathway offers a novel approach to reversing MDR and improving therapeutic outcomes.

Based on the above mechanism, potent Shh antagonist cyclopamine (CYP) shows good potential application value [18]. CYP is a natural steroid alkaloid that exerts inhibitory effects by competitively binding to the transmembrane domain of the Smo receptor [19,20,21]. Clinical studies have found that CYP can inhibit tumor growth in patients with basal cell carcinoma [22]. However, the toxicity to the central nervous system and the poor solubility (<1 mM) of CYP limit its clinical use [23].

Combination therapies, which target multiple pathways simultaneously, offer a more effective strategy for overcoming resistance [24,25]. Recent decades have seen major breakthroughs in scientific innovation [18,26]. On the other hand, nano-delivery systems can enhance the tumor targeting of drugs through the enhanced penetration and retention (EPR) effect, and improve the bioavailability of hydrophobic drugs (such as CYP), and spatial–temporal synchronous release, thus improving the efficacy of antitumor drugs [27,28,29].

Paclitaxel (PTX) is one of the most widely used antitumor drugs, but it is a substrate for MDR [30]. Therefore, combining antitumor PTX and Shh pathway inhibitors with a nano-delivery system that acts on separate targets may achieve therapeutic synergies in breast cancer by overcoming the complexity of MDR.

In this study, we prepared CYP-loaded PLGA-PEG nanoparticles (CYP NPs, 200 nm) and PTX-loaded PLA-PEG nanoparticles (PTX NPs, 30 nm). PTX directly kills tumor cells, while CYP down-regulates P-gp expression by inhibiting the Shh pathway, thereby reducing PTX efflux. We expected that the combination applications would enhance the treatment of drug-resistant tumors through synergistic Shh pathway inhibition and drug accumulation and penetration improvement in tumors. Systematic evaluation was conducted to determine CYP NPs and PTX NPs’ performance in overcoming drug-resistant breast cancer and to understand the mechanism of how the two agents work at the cellular, multicellular spheroid, and animal levels.

## 2. Materials and Methods

### 2.1. Materials

Polyethylene glycol–polylactic acid copolymer (PEG_2000_-PLA_2000_) was purchased from Advanced Polymer Materials Inc. (Montreal, QC, Canada). Polyethylene glycol–poly(lactic-co-glycolic acid) copolymer (PEG_5000_-PLGA_23000_) was purchased from Jinan Daigang Biomaterial Co., Ltd. (Jinan, China, 764779). Paclitaxel (PTX) was obtained from Meilun Technology Co., Ltd. (Dalian, China, MB1178). Cyclopamine (CYP) was purchased from Nanjing Spring & Autumn Biological Engineering Co., Ltd. (Nanjing, China, B20547). All other chemicals were analytical or high-performance liquid chromatography (HPLC) grade.

Rhodamine-123 (R-123), 3-(4,5-dimethylthiazol-2-yl)-2,5-diphenyltetrazolium bromide (MTT), 1,1-dioctadecyl-3,3,3,3-tetramethylindodicarbocyanine (DiD), coumarin-6 (COU, purity > 99%), and verapamil were purchased from Sigma-Aldrich (St. Louis, MO, USA). P-gp antibody (557002, FITC-mouse anti-human P-glycoprotein, BD) and the isotype control antibody (555742, FITC-mouse IgG2b k isotype control, BD) were purchased from Becton Dickinson (San Jose, CA, USA). The one-step TUNEL cell apoptosis detection kit was obtained from Beyotime Biotechnology (Shanghai, China, C1086). The Annexin V-FITC apoptosis detection kit and in situ cell death detection kit were purchased from KeyGEN BioTech Co., Ltd. (Nanjing, China, KGA1406). Anti-Ki67 antibody was purchased from Abcam (EPR3610, Cambridge, UK). Culture plates and dishes were purchased from Corning Inc. (430641, 3516, Corning, NY, USA).

MCF-7 cells and MCF-7/Adr cells were purchased from the Institute of Basic Medical Sciences (Peking Union Medical College, Beijing, China). RPMI-1640 medium, modified eagle medium (MEM), penicillin–streptomycin, and trypsin were obtained from Macgene Technology (Beijing, China). MCF-7 and MCF-7/Adr cells were grown in RPMI-1640 containing 10% fetal bovine serum and 1% antibiotic cocktail (100 U/mL penicillin + 100 μg/mL streptomycin), with incubation at 37 °C under 5% CO_2_.

The female BALB/c nude mice (8 weeks old, 18–20 g) were provided by the Vital Laboratory Animal Center (Beijing, China). All care of the animals was performed under SPF conditions with free access to standard food and water. All care and handling of animals were performed with the approval of the Institutional Animal Care and Use Committee at Peking University Health Science Center.

### 2.2. Preparation of PTX-Loaded PEG-PLA Nanoparticles and CYP-Loaded PEG-PLGA Nanoparticles

PTX-loaded PEG-PLA nanoparticles (PTX NPs) were prepared by the solvent evaporation method [31]. In brief, both PEG-PLA and PTX were dissolved in acetonitrile and evaporated for 30 min at 50 °C. Then, we added phosphate-buffered saline (PBS, pH 7.4) and performed ultrasonic treatment for 2 min, and PTX NPs were gained. The final processing step involved passing the solution through a 0.22 μm sterile filter membrane. DiD-loaded PEG-PLA nanoparticles were prepared with the same procedure, except that PTX was replaced by DiD.

CYP-loaded PEG-PLGA nanoparticles (CYP NPs) were formed by the emulsification–solvent evaporation method [32]. In brief, PEG-PLGA and CYP were co-dissolved in chloroform and added to 5 mL 5% glucose solution containing 2% F68 dropwise at room temperature. The formulation process involved (1) ice-bath-assisted probe sonication (100 W/5 min) using JY92-2D equipment (Ningbo Scientz Biotech, Ningbo, China), followed by (2) 4 min vacuum concentration and (3) final membrane filtration (0.45 μm pore size).

The PTX NPs and CYP NPs were analyzed through a transmission electron microscope (JEM-2100F, JEOL, Japan) and dynamic light scattering analysis (Malvern Zetasizer 3000, Malvern Panalytical Ltd., UK) at 25 °C.

Meanwhile, the PTX injection was prepared as a formulation of 6 mg PTX, 527 mg Cremophor EL, and 49.7% ethanol, and was diluted with PBS. The CYP injectable formulation was formulated through dissolution in dimethyl sulfoxide (DMSO), followed by subsequent dilution with PBS to maintain a final DMSO concentration under 1% (*v*/*v*).

### 2.3. Quantitation of CYP and PTX

The concentration of PTX was monitored by an HPLC system (Shimadzu Corporation, Kyoto, Japan). A reverse-phase C18 column (250 × 4.6 mm, 5 μm, Dikma, Markham, ON, Canada) was used for investigation. The mobile phase was acetonitrile and water (55/45, *v*/*v*) with a flow rate of 1.0 mL/min. The detection wavelength was 227 nm.

CYP quantification was performed using a Dikma C18 reversed-phase column (Platisil ODS, 5 μm, 250 mm × 4.6 mm, Dikma, Markham, ON, Canada). The mobile phase was acetonitrile and 0.03% (*v*/*v*) trimethylamine aqueous solution in a volumetric ratio of 85:15 with a flow rate of 1.0 mL/min. UV detection was conducted at a 210 nm wavelength [33].

The encapsulation efficiency (EE) rates of PTX NPs and CYP NPs were calculated as (W_total drug_ − W_free drug_)/W_total drug_ × 100%. Triplicate measurements were performed for each experimental sample to ensure analytical precision.

### 2.4. P-gp Expression in MCF-7 and MCF-7/Adr Cells

MCF-7 or MCF-7/Adr cells were seeded in 6-well plates at a density of 5 × 10^5^ cells/well. After cultivation at 37 °C for 24 h, cells were washed with PBS and treated with serum-free RPMI 1640 medium containing CYP solution, CYP NPs, PTX NPs, and PTX NPs+CYP NPs for 12 h, respectively. The final concentrations of CYP and PTX were 20 μM and 2.5 μg/mL, respectively. Then, cells were washed with PBS twice, collected, and immobilized with 4% paraformaldehyde. P-gp-FITC and isotype antibody were added to the washed cells and incubated at 4 °C for 90 min. Cells were analyzed via flow cytometry (Becton Dickinson, CA, USA) after being washed with PBS.

### 2.5. Nanoparticle Accumulation in MCF-7 and MCF-7/Adr Cells

The uptake of rhodamine 123 (R-123)-labeled nanoparticles in MCF-7 and MCF-7/Adr cells was assessed with the same procedure as the previous report [34]. MCF-7 and MCF-7/Adr cells (2 × 10^5^) were seeded on a 12-well cell culture plate for 24 h. Then, the cells were washed twice with PBS and cultured with serum-free medium in each well containing free R-123 (1.76 μM) with CYP, CYP NPs, blank nanoparticles, and the P-gp inhibitor verapamil for 2 h. The concentration of CYP and verapamil was 30 μM and 15 μg/mL, respectively. The concentration of blank nanoparticles was the corresponding PLGA concentration of CYP NPs. Following incubation, cellular specimens underwent sequential processing: harvesting via centrifugation, triple rinse with ice-cold PBS, resuspension in 500 μL PBS, and subsequent flow cytometric analysis.

### 2.6. Cytotoxicity Study

The respective and combined cytotoxicity of the CYP and PTX formulations to MCF-7 and MCF-7/Adr cells was assessed by the MTT assay. Briefly, cells in the exponential phase of growth were seeded at a density of 10,000 cells/well in 96-well plates and incubated for 24 h. Then, the cells were incubated with various concentrations of CYP, CYP NPs, blank nanoparticles, PTX NPs or combinations of CYP NPs and PTX NPs for 48 h. Following incubation at 37 °C for 4 h, the culture medium was carefully removed and replaced with 200 μL DMSO per well. This procedure was preceded by the addition of 20 μL MTT solution (5 mg/mL concentration) to each well. The absorbance measurements were subsequently performed at a 570 nm wavelength using a BioRad iMark microplate absorbance reader.

### 2.7. Cell Apoptosis Analysis

MCF-7/Adr cells were seeded in 6-well plates at a density of 5 × 10^5^ cells/well and cultured at 37 °C for 24 h. Afterwards, cells were washed with PBS and incubated with PTX NPs, CYP NPs and PTX NPs+CYP NPs for 24 h. The final concentration of CYP and PTX was 30 μM and 2.5 μg/mL, respectively. The cells incubated with serum-free RPMI 1640 medium were treated as a control. After incubation, all the cells were collected and washed twice with cold PBS, resuspended in the binding buffer, and stained with Annexin V-FTIC and PI according to the manufacturer’s protocol. The stained samples were analyzed by flow cytometry immediately. Cell Quest 3.3 software (Becton Dickinson, CA, USA) was used to analyze the data.

### 2.8. Penetration of Three-Dimensional Multicellular Tumor Spheroids

We used MCF-7/Adr tumor spheroids model to imitate the environment of the drug-resistant tumor. R-123, as a fluorescence agent and P-gp subtract, was used to simulate the behavior of chemotherapeutic drugs in the tumor site after CYP treatment. In brief, MCF-7/Adr spheroids were prepared through the hanging drop method, which was the same as the reported method [35]. When the diameters of the spheroids were about 250 μm, the spheroids were incubated with R-123 (control), R-123+CYP and R-123+CYP NPs for 24 h. The final concentrations of R-123 and CYP were 10 μM and 30 μM, respectively. Following PBS rinsing, the tumor spheroids were relocated to chambered coverslips for microscopic examination using laser scanning confocal microscopy. Sequential optical sections were acquired through axial scanning from the spheroid base to its central region, with 5 μm intervals between adjacent imaging planes. Meanwhile, the 3D integrated pictures were obtained from the single-layered scan images.

### 2.9. In Vivo Antitumor Efficacy

The antitumor efficacy in vivo was conducted in MCF-7/Adr tumor-bearing female Balb/c nude mice. Briefly, 7.5 × 10^6^ MCF-7/Adr cells were subcutaneously inoculated into the right flank of the mice. The tumor size reached about 100 mm^3^ after 27 days, and that day was marked as Day 0. Afterwards, mice were randomly divided into four groups (5 mice/group). Afterwards, mice were intravenously injected with PTX NPs and peritumorally injected with CYP NPs, PTX NPs+CYP NPs, and physiological saline (control), respectively, on Days 1, 4, and 7. The dose of PTX and CYP was 8 mg/kg and 10 mg/kg, respectively. The body weight and the tumor volume of mice were carefully recorded every day. The tumor volumes (*V*) were calculated using the equation *V* = 0.5 × *a* × *b*^2^, with *a* and *b* standing for the longest diameter and shortest diameter, respectively. Mice were euthanized on Day 16, and the tumor, heart, liver, spleen, lungs, and kidneys were harvested, rinsed and weighed. Tumor tissue was frozen using O.C.T. embedding medium and sectioned into 4 mm thick slices. The frozen sections were detected by TUNEL and Ki-67 assays.

### 2.10. TUNEL Assay

Apoptotic nuclei in frozen tissue specimens were identified through dUTP nick-end labeling (TUNEL) utilizing a one-step TUNEL cell apoptosis detection kit, with fluorescent signal acquisition and quantification executed under CLSM imaging parameters.

### 2.11. Ki-67 Assay

To observe the proliferation condition of the tumor tissue, frozen sections were fixed with 4% formaldehyde. After being blocked with 2% BSA, the sections underwent sequential incubation with anti-Ki-67 primary antibody, a fluorescent secondary antibody, and Hoechst 33258 nuclear counterstain under standardized immunolabeling conditions. All frozen sections were analyzed using CLSM.

### 2.12. Perfusion and Microvessel Density Observation

The perfusion analysis was conducted similarly to the reported study [36]. On Day 16, mice were injected with 100 μg/kg DiD-loaded PEG-PLA nanoparticles. After injection for 5 h, mice were intravenously injected with 10 mg/mouse FITC–dextran solution. Three hours after injection, mice were sacrificed and tumors were removed for frozen sections. The red light (DiD-loaded PEG-PLA nanoparticles) and green light (FITC-dextran) of the samples were observed by confocal microscopy.

### 2.13. Blood Test

Four blood samples of each group were collected on Day 16. The white blood cells (WBCs), red blood cells (RBCs), platelets (PLT), lymphocytes (LYM), and neutrophils (GRN) were analyzed.

### 2.14. Statistical Analysis

Either a Student’s t-test or a one-way analysis of variance (ANOVA) was used to evaluate the data, and the software packages GraphPad Prism 9.0 and SPSS 24.0 were used to analyze the data. A *p*-value less than 0.05 was considered statistically significant. * *p* < 0.05, ** *p* < 0.01, *** *p* < 0.001.

## 3. Results and Discussion

### 3.1. Characterization of CYP NPs and PTX NPs

PEG-PLA and PEG-PLGA are widely used and reliable biodegradable polymers that have been approved by the Food and Drug Administration (FDA) for multiple drug delivery and biomedical device applications [37,38,39,40]. Polymeric micelles or nanoparticles from PEGylated block copolymers have been widely studied in preclinical and clinical trials [41,42]. The dense PEG shell provides a stealth coating, allowing the nano-drug delivery system to evade being captured by the macrophage and prolong its circulation time in vivo [43,44].

**Figure 1:**
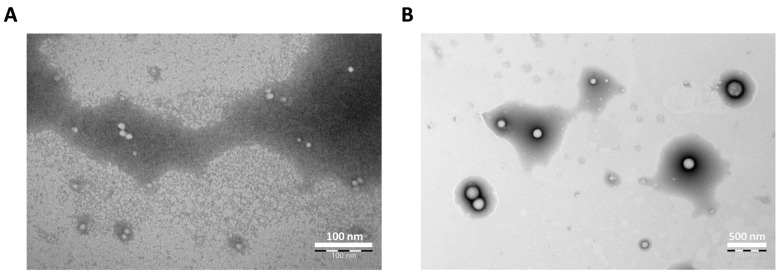
Photograph of (**A**) PTX NPs (scale bar 100 nm) and (**B**) CYP NPs (scale bar 500 nm) as determined by transmission electron microscopy.

In this study, we prepared CYP NPs and PTX NPs by using PEG-PLGA and PEG-PLA, respectively. Dynamic light scattering (DLS) analysis (Table 1) showed the CYP NPs had a larger size of 200 nm in diameter, and the PTX NPs had a smaller size of 30 nm in diameter. Both nanoparticles are spherical as illustrated by transmission electron microscopy (TEM) (Figure 1A,B). The encapsulation efficacy rates of the two drugs were both above 90%.

It is well known that smaller (sub-100 nm) nanoparticles possess a greater extent of extravasation and/or penetrate further away from the vasculature than larger systems [45,46,47], while nanoparticles with larger sizes show higher retention in tumors [48,49]. Therefore, we consider that the CYP NPs (200 nm) enable the down-regulation of P-gp expression and inhibit its activity durably due to their greater retention, while the PTX NPs (30 nm) are inclined to penetrate into tumors by relying on the small particle size to kill cancer cells.

### 3.2. Repressive P-gp Expression and Enhanced P-gp Substrate Internalization in Resistant Cells by CYP Formulations

**Figure 2:**
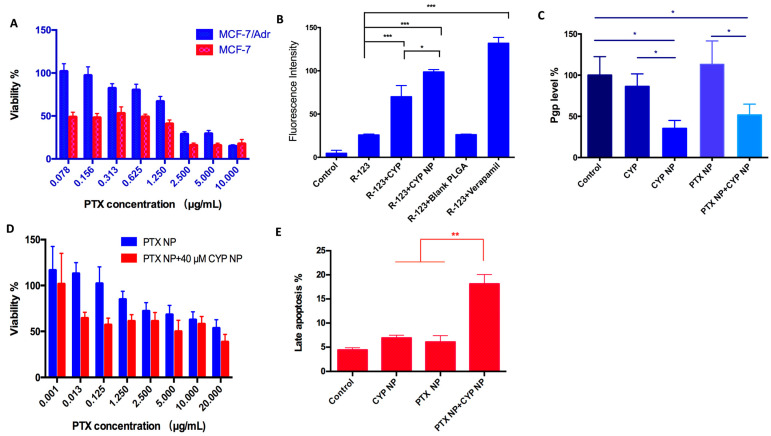
(**A**) Survival rate of MCF-7 cells and MCF-7/Adr cells cultured with various concentrations of PTX solution for 48 h (*n* = 6). There were substantial differences (*p* < 0.01) between the two cell lines. (**B**) Internalization of R-123 by MCF-7/Adr cells after applying different CYP formulations (*n* = 3). (**C**) P-gp level of MCF-7/Adr cells after being treated with PBS (as control), CYP, CYP NPs, PTX NPs, and PTX NPs+CYP NPs. The final concentrations of CYP and PTX are 20 μM and 2.5 μg/mL, respectively (*n* = 3). (**D**) Survival rate of MCF-7/Adr cells after being cultured with different treatments for 48 h (*n* = 6). (**E**) Cell apoptosis evaluation of PBS (as control), CYP NPs, PTX NPs, and PTX NPs+CYP NPs against MCF-7/Adr cells for 24 h (*n* = 3). All the data are presented as the mean ± SD. * *p* < 0.05, ** *p* < 0.01, *** *p* < 0.001.

Tumor cells become resistant to chemotherapy mostly because of P-gp transporter overexpression in cells, which efflux drugs [50]. The positive percentages of P-gp expression in MCF-7 and MCF-7/Adr cells were 43.37% and 64.34%, respectively (Figure S1). To further verify that MCF-7/Adr cells were drug-resistant, a proliferation assay of PTX in MCF-7 and MCF-7/Adr cells was conducted. There were significant differences between the viability of the two cell lines (*p* < 0.01, Figure 2A). The resistance index (IC_50 MCF-7/Adr_/IC_50 MCF-7_) was calculated as 10.7, demonstrating that MCF-7/Adr cells were highly resistant to PTX, whereas the blank PEG-PLA nanoparticles showed negligible inhibition on both MCF-7 and MCF-7/Adr cells in vitro (Figure S2), which implied the good biocompatibility of the material. The cytotoxicity of the SHH inhibitor CYP was far weaker than that of the antitumor drug PTX (Figure S3). When the concentration of CYP NPs was 40 μM, the viability of MCF-7/Adr cells reached 60%. CYP NPs were a little more toxic than CYP, which was associated with the fast uptake of nanoparticles.

Drug-resistant cells inhibit the internalization of drugs in the cells, and drugs activate the ABC transporters on the cell membrane, resulting in the pumping out of the drugs that reach the cytoplasm. Rhodamine-123 (R-123) is a P-gp fluorescent substrate [51] and is usually used to examine the membrane transport ability of P-gp [52]. To assess if CYP or the CYP NPs have an inhibitory effect on P-gp activity, R-123 was added with free CYP, CYP NPs, and blank nanoparticles to MCF-7/Adr cells. Verapamil, a P-gp inhibitor, acted as the positive control group. The internalization of R-123 in MCF-7/Adr cells is summarized in Figure 2B. The internalization of free R-123 in MCF-7/Adr cells was very low, which was due to the fast efflux activity via P-gp. The blank nanoparticles had no influence on the uptake of R-123, which demonstrated that materials had no influence on P-gp activity. The R-123 internalization level significantly increased in the presence of CYP, CYP NPs, and verapamil (3.1-fold, 4.4-fold, and 6.0-fold, respectively). Obviously, the encapsulation of CYP into nanoparticles (CYP NPs) can improve drug internalization in resistant cells when compared with free CYP. The phenomenon was attributed to the inhibiting effect of CYP and verapamil on P-gp, implying that CYP has the ability to prevent drug efflux.

We compared the ability of CYP or/and CYP NPs to down-regulate P-gp expression in MCF-7/Adr cells. After 24 h treatment with various formulations, the CYP NPs were significantly more effective than the free CYP at down-regulating P-gp expression levels (Figure 2C). PTX NPs had no obvious influence on P-gp expression levels, while the combination group (CYP NP + PTX NP) reduced P-gp levels to a similar extent to CYP NPs alone. It is reported that the up-regulation of MDR1 and BCRP expression in drug-resistant cells by Shh pathway overexpression may be a reason for tumor drug resistance [53]. We infer that the inhibition of the Shh pathway by CYP and CYP NPs may lead to the down-regulation of MDR1; therefore, P-gp, which is the downstream protein of MDR1, would decrease and overcome drug resistance. The more down-regulated expression of P-gp by CYP NPs (Figure 2C) is in accordance with its increased internalization into cells (Figure 2B). CYP NPs promoted the uptake of PTX into the cells and hence concentrated the drugs in tumor cells. Down-regulating P-gp expression and inhibition of the Shh pathway by CYP NPs reversed the drug-resistant cells to drug-sensitive cells, which made the tumor treatable.

The increased cytotoxicity of PTX NPs to MCF-7/Adr cells when combined with CYP NPs was shown in Figure 2D. The IC_50_ of PTX NPs was decreased from 25.33 μg/mL to 16.04 μg/mL after the addition of 40 μM of CYP NPs. This highlights that the addition of CYP NPs strongly enhanced the sensitivity of MCF-7/Adr cells to PTX NPs. Thus, CYP NPs are a promising antitumor enhancer for combination therapy with PTX.

Beyond inhibiting cancer cell proliferation, apoptosis induction in cancer cells is another promising strategy for cancer treatment. Therefore, an apoptosis assay was conducted when CYP NPs and PTX NPs were cultured with MCF-7/Adr cells for 24 h (Figure 2E). The results established that both CYP NPs and PTX NPs possessed the ability to induce apoptosis, and the combination of CYP NPs with PTX NPs increased the late apoptosis percentage to more than twice that of each one alone. It is known that Shh has an effect on regulating cell cycle progression and apoptosis resistance [54,55]. By blocking the Shh pathway, CYP NPs were able to overcome the effect of apoptosis resistance of tumor cells caused by overexpression of Shh. All these results showed that P-gp in MCF-7/Adr cells was suppressed by CYP NPs, and the efflux of PTX was down-regulated, resulting in the accumulation of PTX in the cytoplasm and more apoptotic cells. Collectively, it can be concluded that CYP has a remarkable inhibitory effect on P-gp efflux activity, and the nanoparticles could enhance its function. The CYP NPs increased the internalization of P-gp substrate in resistant cells and subsequently increased proliferation and apoptosis.

### 3.3. Enhanced P-gp Substrate Penetration in Resistant Tumor Spheroids by CYP Formulations

**Figure 3:**
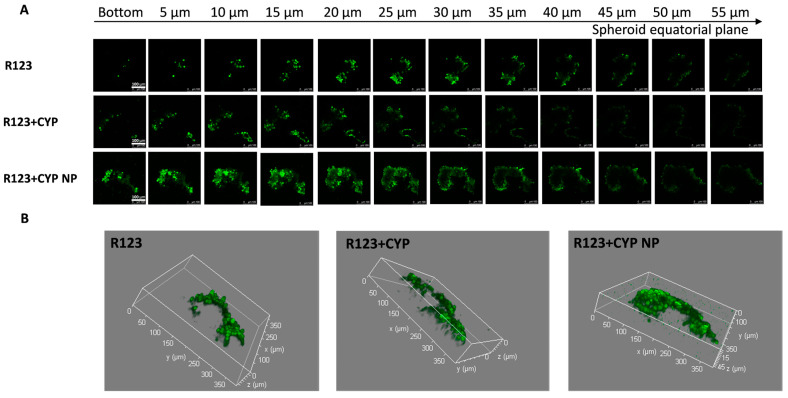
(**A**) Confocal Z-stack images of MCF-7/Adr tumor spheroids and (**B**) 3D integrated model after incubation with various R-123 solutions, R-123+CYP, R-123+CYP NPs for 24 h, respectively. Z-stack images were attained from the bottom towards the tumor spheroid equatorial plane at a 5 μm thickness. The scale bar represents 100 μm.

Limited penetration and accumulation are two of the prominent factors leading to solid tumor treatment failure [56,57]. Two-dimensional cell culture studies have limitations in evaluating the penetration and accumulation of drugs in solid tumors with a complex structure and composition. MCF-7/Adr spheroids were used to simulate the microenvironment of solid tumors and were treated with CYP formulations. The effect of CYP NPs and CYP solution on the penetration of the P-gp substrate was observed by confocal microscopy. Images were captured from the bottom to the equatorial plane of the spheroids and 3D integrated models were also assembled (Figure 3).

R-123 in the control group was retained mainly on the surface of the spheroids; the high expression of P-gp on MCF-7/Adr cells counteracted the diffusion and access of drugs. The low-concentration CYP was inefficient in P-gp inhibition in the deeper layers of the spheroids, and R-123 in the CYP solution-treated group was also retained mainly on the surface of the spheroid. The permeation ability of R-123 was significantly enhanced after the co-administration of CYP NPs, indicating that CYP NPs have a better effect on promoting the tumor penetration of the P-gp substrate drugs. Studies have shown that nanoparticles could enhance the cellular uptake of encapsulated drugs, and their diffusion plays an important role in the penetration of nanoparticles into the tissue matrix [58,59]. The relatively large particle size (200 nm) allows the CYP NPs to stay in the tumor after they enter the tumor. The CYP NPs played an essential part in the penetration and accumulation abilities in tumors and inhibition of P-gp activity, leading to the deeper permeation of R-123. Based on the fluorescent probe penetration results, we speculated that CYP NPs could enhance other P-gp substrates, such as PTX, to accumulate in the solid tumor.

### 3.4. Therapeutic Efficacy in Resistant Breast Cancer Xenograft Mice

**Figure 4:**
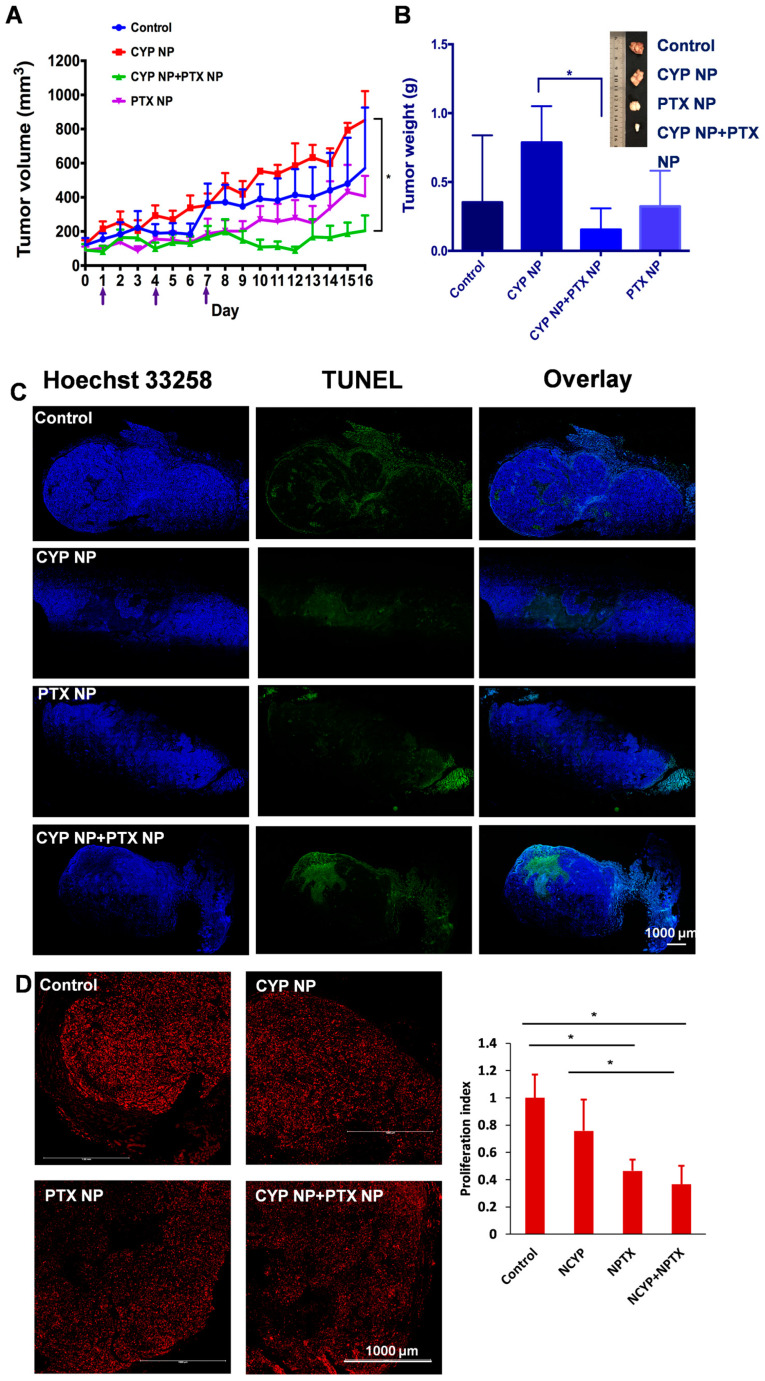
(**A**) Tumor volume–time curve of female MCF-7/Adr-bearing mice treated every three days for three i.v. injections of PTX NPs, peritumoral injections of CYP NPs, or saline control (*n* = 6 for control and CYP+PTX NP groups, *n* = 5 for CYP NP and PTX NP groups, arrows indicate the administration time). (**B**) Tumor tissues were separated and weighed on Day 16 after treatment with different formulations (*n* = 5 or 6). (**C**) Cell apoptosis by TUNEL staining and (**D**) proliferation by Ki-67 staining of the dissected tumor tissue after being treated with saline, CYP NPs, PTX NPs and PTX NPs+CYP NPs in vivo, respectively. Apoptotic cells were stained by TUNEL (green) and cell nuclei were stained with Hochest 33258 (blue). Ki-67-positive nuclei were red. The Ki-67-positive cell nuclei proportion relative to the total cell population defined the proliferation index. The results are presented as the mean ± SD. * *p* < 0.05.

The antitumor effect of PTX NP and CYP NP combination therapy was assessed in MCF-7/Adr-bearing female nude mice. Saline, CYP NPs, PTX NPs, and CYP NPs+PTX NPs were administered by three injections at two-day intervals when tumor sizes reached about 100 mm^3^, respectively. CYP NPs were peritumorally injected at a dose of 10 mg/kg and PTX NPs were intravenously injected at a dose of 8 mg/kg. The tumor volume–time curve and tumor weight are revealed in Figure 4. The tumor volume of CYP NP treatment mice grew fast, in accordance with its negligible inhibition of tumor cells in vitro (Figure S3). For PTX NP treatment mice, the speed of tumor growth became slower. The treatment of CYP NPs+PTX NPs kept the tumor volumes of the mice almost unchanged (Figure 4A). Tumor weight results (Figure 4B) corresponded with tumor volume results. This highlights that combination therapy was effective in drug-resistant tumors in vivo. PTX-treated mice exhibited varying degrees of TUNEL staining-positive cells, and CYP NP+PTX NP-treated mice had the largest number of apoptotic cells (Figure 4C). This result is also relevant to the results of the apoptosis experiment (Figure 2E). The CYP NP+PTX NP treatment mice displayed the lowest Ki-67-positive nuclei and the lowest proliferation index among these four groups (Figure 4D). It highlights that the combination of CYP NPs with PTX NPs displays outstanding apoptosis and proliferation inhibition in vivo. We believe that these outcomes are due to the combination of the two nanoparticles, which achieve synergistic inhibition of the Shh pathway by CYP, boost PTX accumulation and penetration in the tumor, and ultimately improve the treatment of drug-resistant cancers. In addition, there was necrosis in the center of the tumor caused by hypoxia and nutrient shortage in the control and CYP NP groups.

### 3.5. Enhanced Biodistribution and Blood Perfusion of Nanoparticles in Tumor by CYP Formulation

**Figure 5:**
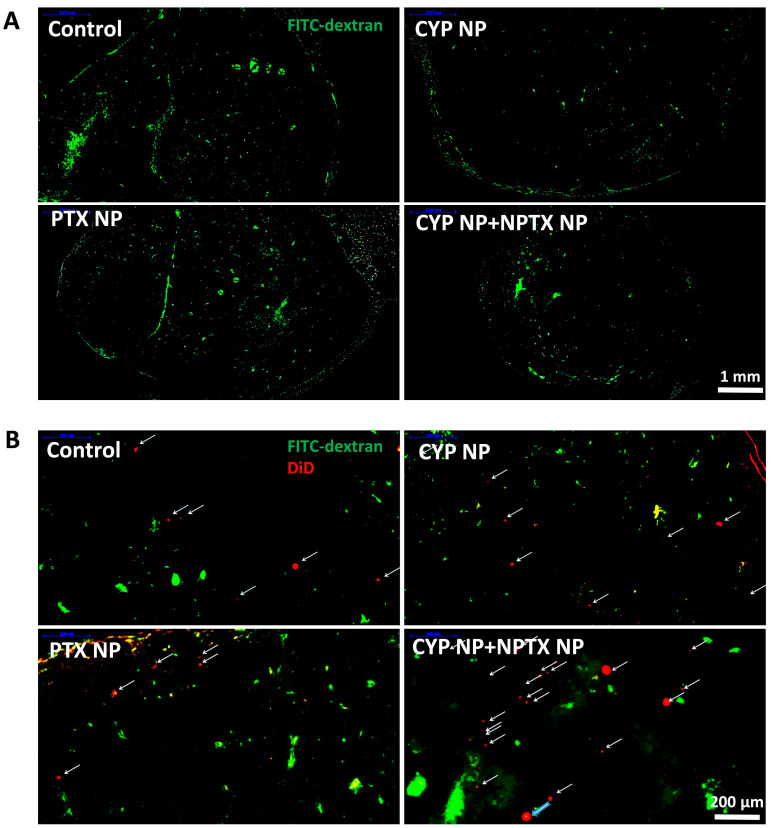
(**A**) Blood perfusion and (**B**) distribution of PTX NPs in tumor tissue treated with FITC–dextran (MW 500,000 Da) and DiD-labeled PEG-PLA nanoparticles in MCF-7/Adr tumors after treatment with saline (control), CYP NPs, PTX NPs, and PTX NPs+CYP NPs in vivo, respectively. DiD-labeled nanoparticles (labeled with arrows) in the magnified area of the slices.

We next compared the biodistribution of nanoparticles and blood perfusion in different treatment groups. Mice from each group after treatment on Day 16 were intravenously injected with 100 μg/kg DiD-loaded PEG-PLA nanoparticles (representative small PTX NPs, 30 nm) and then intravenously injected with 10 mg/mouse FITC–dextran solution 5 h later. FITC–dextran (MW 500,000 Da) can adhere to the surface of the endothelial cells and then be absorbed into or retained in the vascular cavity as a perfusion indicator [60]. Therefore, the blood perfusion (Figure 5A) and distribution of PTX NPs in tumor tissue (Figure 5B) can be observed indirectly by laser scanning confocal microscopy. From the confocal images, compared with the control group, green fluorescence (FITC represents blood perfusion) and red fluorescence (DiD represents nanoparticles) in CYP NP-treated groups and the CYP NP+PTX NP group increased significantly. This highlights that small nanoparticles can reach the tumor site, and CYP NPs enhance blood perfusion, which contributes to reduced tumor angiogenesis and decreased matrix components [61].

The effective accumulation and penetration of chemotherapeutic drugs in solid tumors are essential in chemotherapy. The application of nanotechnology in the delivery of PTX NPs brought favorable biodistribution profiles and prolonged systemic circulation lifetime. Specifically, PTX NPs took advantage of the EPR effect and were retained in the tumor tissue due to their small size. The peritumoral injection of CYP NPs possessed a long-lasting effect in inhibiting and down-regulating MDR, which permitted the intracellular accumulation and activity of PTX. The elimination of the tumor matrix by CYP NPs further increases the enrichment and penetration of PTX NPs in deep tumor areas for anticancer efficacy.

### 3.6. Safety Assay

**Figure 6:**
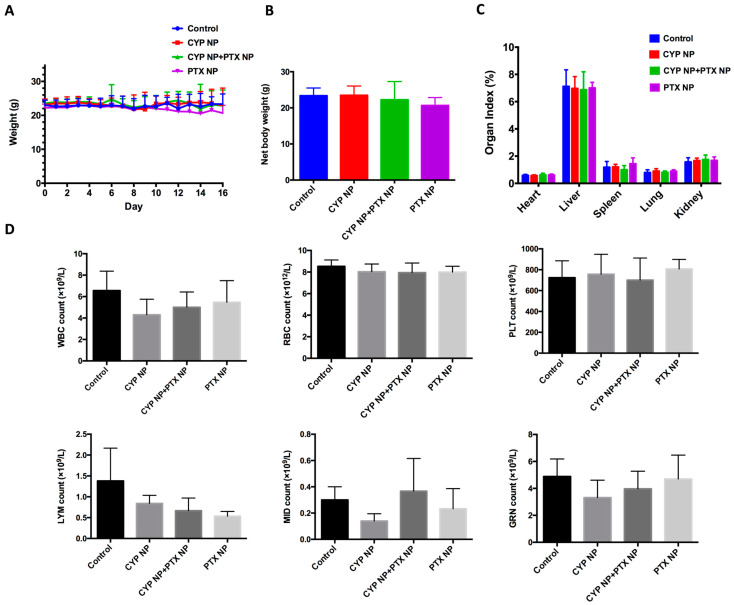
(**A**) Body weight, (**B**) net body weight and (**C**) organ index after treatment with different formulations in the tumor-bearing mice (*n* = 6 for control and CYP NP+PTX NP groups, *n* = 5 for CYP NP and PTX NP groups). (**D**) Hematological indicators including WBCs, RBCs, PLT, LYM and GRN on Day 16 (*n* = 4). The results are presented as the mean ± SD.

The superior antitumor capacity of the combination of CYP NPs and PTX NPs motivated us to further evaluate its toxicity profile. Figure 6A demonstrates the body weights of mice after treatment, showing that mice in all groups did not suffer significant body weight loss. Net body weight (Figure 6B) and organ index (Figure 6C) of all groups also did not show a remarkable difference compared to the control group. As PTX may lead to myelosuppression [62], hematological effects were tested by routine peripheral blood examination (Figure 6D) on Day 16 (the 9th day after the last drug administration). There was no significant difference compared to the control group in the counts of WBCs, RBCs, PLT, LYM and GRN in routine peripheral blood tests. According to the results, we determined that PTX NPs, CYP NPs and PTX NP+CYP NP combination administration were relatively safe. This lower toxicity profile is in line with the good biocompatibility of the carrier material and negligible cytotoxicity of blank nanoparticles in the MTT assay (Figure S2). In brief, in the MCF-7/Adr xenograft model, the combination of CYP NPs and PTX NPs showed enhanced efficacy against drug-resistant tumors with lower systemic toxicity.

## 4. Conclusions

Activation of the Shh signaling pathway has been implicated in up-regulating P-gp expression, a key mediator of MDR that compromises chemotherapeutic efficacy through drug efflux mechanisms. We use CYP as a potent Shh pathway antagonist through competitive binding to the Smo receptor, effectively blocking downstream pathway activation. This Shh inhibition strategy was synergistically combined with PTX chemotherapy to develop a dual-action therapeutic approach. The implementation of nanotechnology addressed two critical limitations: poor aqueous solubility of both CYP and PTX, which traditionally restricts bioavailability, and enhanced tumor-targeted delivery through the EPR effect. Our engineered CYP nanoparticles (~200 nm) demonstrated prolonged intratumoral retention, enabling sustained Shh pathway inhibition and P-gp functional suppression. This size-optimized formulation created a favorable tumor microenvironment for the smaller PTX nanoparticles (~30 nm), facilitating deeper tumor penetration and enhanced cellular uptake. Meanwhile, by down-regulating P-gp expression by about 50%, CYP NPs could convert drug-resistant cells to PTX-sensitive cells in both cytotoxicity and apoptosis induction through the Shh pathway. The in vivo study demonstrated synergistic therapeutic outcomes. CYP NPs+PTX NPs showed tumor growth inhibition in approximately 78% and PTX NPs alone in approximately 42%. There was no significant weight loss and no significant difference in hematology, suggesting that CYP NPs in combination with PTX NPs synergistically maximize their therapeutic efficacy and safety. It is convincing that a combination of Shh pathway inhibitors and chemotherapeutics is a promising strategy in cancer therapy.

## Figures and Tables

**Table 1:** Characteristics of PTX NPs and CYP NPs measured by DLS. Data are presented as the mean ± SD (*n* = 3).

	Size (nm)	Polydispersity Index	Zeta Potential (mV)	Encapsulation Efficiency (%)
PTX NP	28.36 ± 6.68	0.245 ± 0.06	−9.82 ± 5.63	90.56 ± 2.30
CYP NP	201.52 ± 31.95	0.228 ± 0.05	−18.63 ± 4.8	98.24 ± 0.41

## Data Availability

The original contributions presented in this study are included in the article/supplementary material. Further inquiries can be directed to the corresponding author.

## Supplementary Materials

The following supporting information can be downloaded at https://www.mdpi.com/article/10.3390/pharmaceutics17050572/s1, Figure S1. The P-gp expression level of MCF-7 cells (A) and MCF-7/Adr (B). Figure S2. Survival rate of MCF-7 cells and MCF-7/Adr cells cultured with the blank PEG-PLA nanoparticles for 48 h (*n* = 6). Figure S3. Survival rate of MCF-7/Adr cells and MCF-7 cells cultured with different concentrations of CYP NP and CYP solution for 48 h (*n* = 6).
