# Supplementary material for: Combination Therapy for Overcoming Multidrug Resistance in Breast Cancer Through Hedgehog Signaling Pathway Regulation
Source: Pharmaceutics. 2025 Apr 26;17(5):572. doi: 10.3390/pharmaceutics17050572 (PMC12114799; doi:10.3390/pharmaceutics17050572)
Supplement: Supplementary file 1 [file pharmaceutics-17-00572-s001.zip › pharmaceutics-3546626-supplementary.pdf]

pharmaceutics

MDPI

Supplementary Materials: Combination Therapy for Overcoming Multidrug Resistance in Breast Cancer Through Hedgehog Signaling Pathway Regulation

Yujie Liu, Yiliang Yang and Xianrong Qi

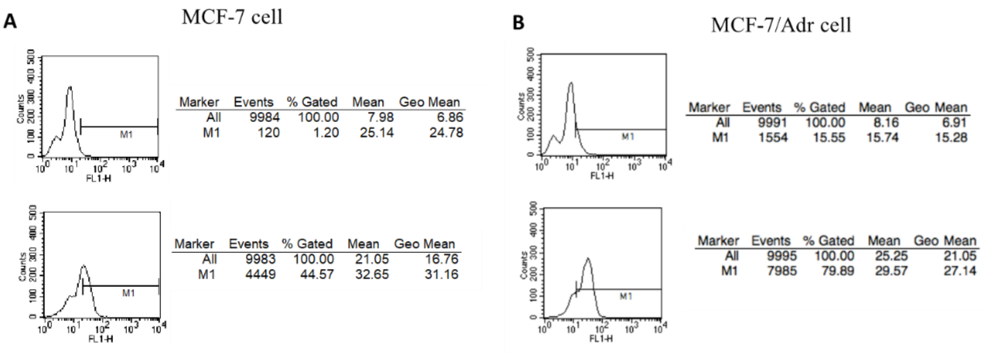

Figure S1. The P-gp expression level of MCF-7 cells (A) and MCF-7/Adr (B).

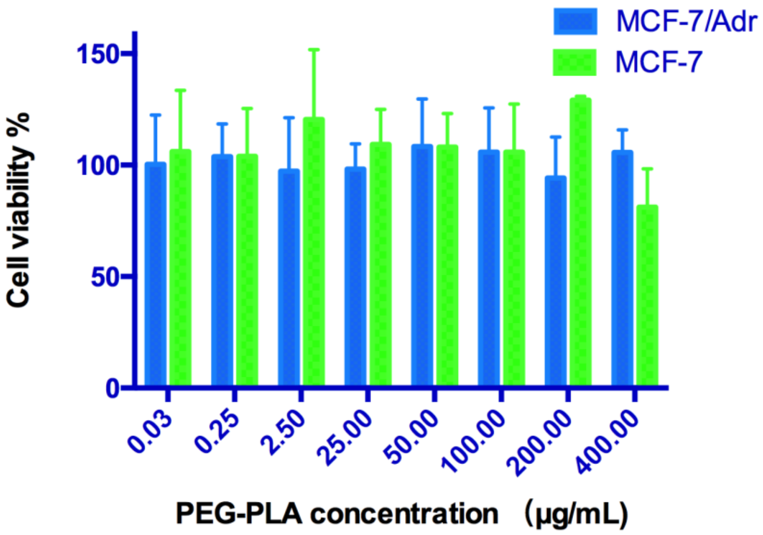

Figure S2. Survival rate of MCF-7 cells and MCF-7/Adr cells cultured with the blank PEG-PLA nanoparticles for 48 h ($n = 6$).

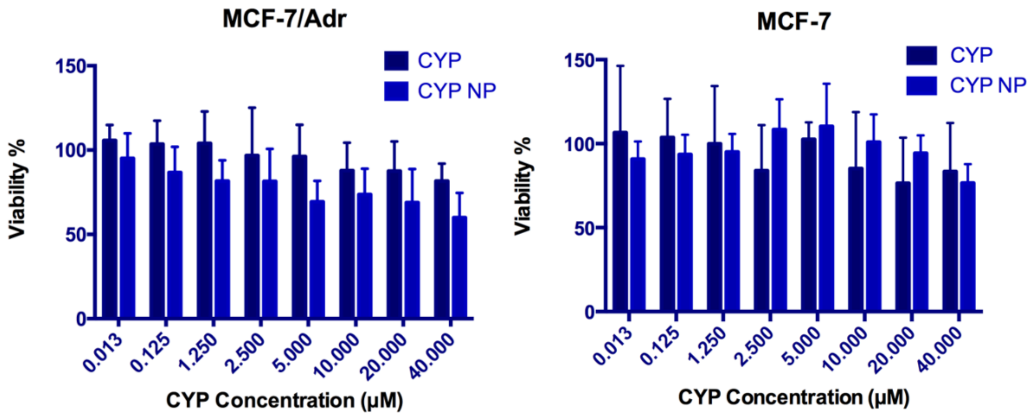

Figure S3. Survival rate of MCF-7/Adr cells and MCF-7 cells cultured with different concentrations of CYP NP and CYP solution for 48 h (n = 6).

Methods

TEM observation: The prepared nanoparticles were dispersed ultrasonic uniformly in deionized water. Take the carbon-supported electron microscope copper mesh, place it on a clean filter paper face up, drop the nanoparticle dispersion solution about 20 μL in the center of the copper mesh, and slowly disperse it in a droplet shape. After the sample was dried, the electron microscope sample rod was removed, the copper mesh was lightly placed on the sample tank, the sample was fixed, and the sample was injected into the Transmission electron microscope (TEM). Set the electron microscope parameters, select a suitable field of view to observe the sample and take a focused photo.
